# Utilization of Phytic Acid as a Selective Depressant for Quartz Activated by Zinc Ions in Smithsonite Flotation

**DOI:** 10.3390/molecules28145361

**Published:** 2023-07-12

**Authors:** Mengtao Wang, Saizhen Jin

**Affiliations:** 1 School of Minerals Processing and Bioengineering, Central South University, Changsha 410083, China; wang.mt@foxmail.com; 2 Faculty of Land Resource Engineering, Kunming University of Science and Technology, Kunming 650093, China

**Keywords:** smithsonite, quartz, flotation separation, zinc ions, phytic acid

## Abstract

It is difficult to separate smithsonite from quartz with metal ion activation through flotation using sodium oleate (NaOL) as the collector. The inevitable Zn^2+^ in the flotation process of zinc oxide ore makes the separation of smithsonite and quartz more difficult. Thus, this study investigated the use of phytic acid (PA) as a flotation depressant to separate smithsonite from Zn^2+^-activated quartz while utilizing sodium oleate as the collector. Microflotation tests indicated that phytic acid could selectively inhibit the flotation of Zn^2+^-activated quartz without affecting the flotation of smithsonite. The measured zeta potentials revealed that the existence of phytic acid hindered sodium oleate adsorption to the surface of Zn^2+^-activated quartz but had little influence on the adsorption of smithsonite. Zn^2+^ dissolution tests and scanning electron microscopy coupled with energy-dispersive X-ray spectroscopy analysis indicated that the phytic acid could dissolve the Zn^2+^ from the minerals’ surfaces into the solution. In conjunction with X-ray photoelectron spectroscopy results, the analysis indicated that phytic acid could adsorb onto the Zn^2+^-activated quartz surface and eliminate active sites for sodium oleate adsorption by dissolving the active Zn^2+^ from the quartz surface into the solution.

## 1. Introduction

Zinc, the fourth most-used non-ferrous metal in the world [1], is utilized primarily in galvanizing, alloys, batteries, and other applications; it is mainly deposited in sulfide and oxidized ores [2,3]. The mining and recovery of oxide ore resources have taken on tremendous importance as a result of the depletion of sulfide ore resources [4]. There are many kinds of zinc oxide ores, including smithsonite, hemimorphite, and zincite, among which smithsonite (ZnCO_3_) is the most common mineral [5,6]. Zinc oxide minerals commonly associate with gangue minerals containing silicon in the deposits, and quartz is one of the widely distributed silicate minerals and major rock-forming minerals in the Earth’s crust [7].

**Figure 1:**
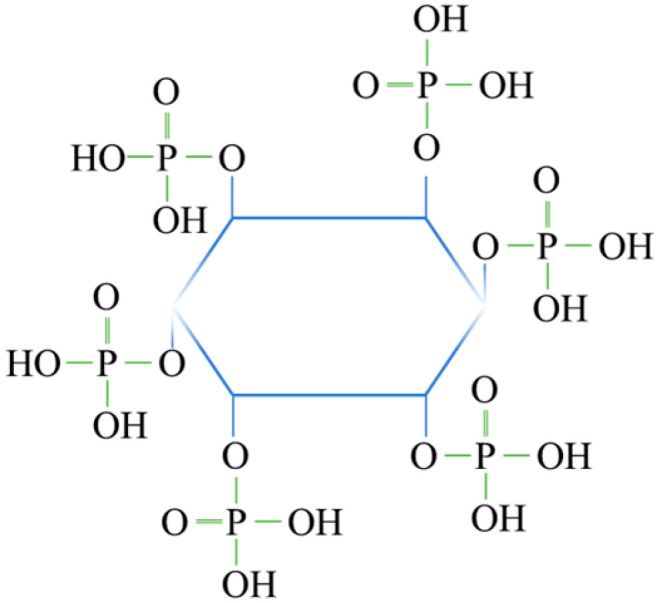
Structural pattern of phytic acid.

Flotation is one of the common methods used to recover smithsonite. Sodium oleate (NaOL) is usually used as the collector for the beneficiation of smithsonite from quartz, and this collector is used successfully in industrial applications [8,9]. However, the presence of a wide variety of gangue minerals and unavoidable ions makes the flotation process of smithsonite and quartz difficult [7,10]. It is easy to separate quartz from smithsonite using NaOL as the collector. However, the flotation of quartz is easily activated by metal ions [8,11,12,13]. When quartz is activated by unavoidable ions, the flotation separation of the minerals becomes challenging. The surface of quartz is negatively charged in a weakly alkaline condition [14]; therefore, cations can adsorb onto its surface and increase the active site for the adsorption of sodium oleate. The floatability of quartz is improved by cations. Previous studies [15] have shown that Mg^2+^ can activate the flotation of quartz, increasing the flotation recovery of quartz to more than 90%, when sodium oleate is used as the collector. Zinc ions also can activate the flotation of quartz, and it is inevitable that zinc ions dissolve into the slurry during the grinding and flotation processes of zinc oxide ores [8,16]. The dissolved zinc ions can improve the floatability of quartz and cause difficulties in the separation of quartz and smithsonite. Depressants are needed to inhibit the flotation of ion-activated quartz [8,10], and it has been reported that sodium silicate, sodium polyaspartate, sodium hexametaphosphate, and some polymers, such as calcium lignosulphonate, carboxymethyl cellulose, and starch, can inhibit the flotation of ion-activated quartz. However, the selectivity of these depressants is poor, and they also inhibit the flotation of smithsonite. Moreover, the dissolution process of macromolecular depressants is difficult to operate. Therefore, the separation of smithsonite from quartz may greatly benefit from researching a more potent depressant with good selectivity. Phytic acid (see Figure 1) is an organic phosphorus compound extracted from plant seeds [17,18]. As a chelating agent, phytic acid can form complexes with calcium, iron, magnesium, zinc, and other metal ions; it is extensively used in the food, pharmaceutical, paint coating, daily chemical, metal processing, textile, plastic, and polymer industries.

In this study, it was planned to use sodium oleate to float smithsonite, while phytic acid would be used to inhibit the flotation of Zn^2+^-activated quartz. The floatability of quartz and smithsonite at various reagent settings was studied using microflotation tests. To study the selective interaction mechanism, the zeta potentials of quartz and smithsonite were measured, the dissolution of zinc ions from the minerals’ surfaces was detected, the micrographs of quartz were captured by SEM, and the chemical states of quartz were analyzed by XPS. 

## 2. Results and Discussion

### 2.1. Effect of Phytic Acid on the Microflotation of Zinc-Ion-Activated Quartz and Smithsonite

**Figure 2:**
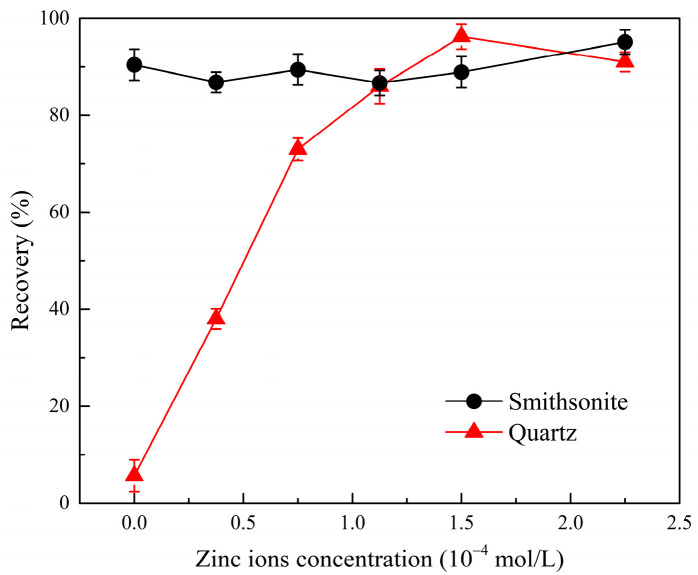
Recovery of smithsonite and quartz at various Zn^2+^ dosages, (C_(NaOL)_ = 120 mg/L, pH = 10).

**Figure 3:**
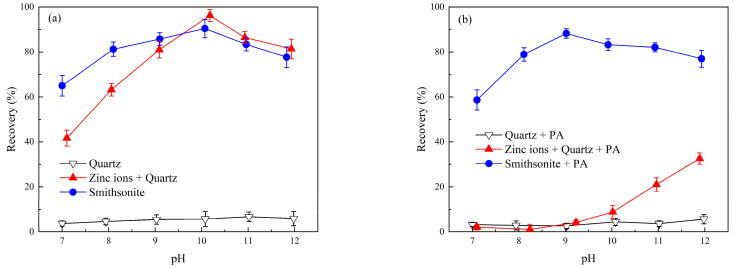
Flotation recovery of smithsonite and quartz at various pH values. (**a**) Without the addition of phytic acid; and (**b**) with the addition of phytic acid, (C_(PA)_ = 35 mg/L, C_(NaOL)_ = 120 mg/L, C_(Zn ions)_ = 1 × 10^−4^ mol/L).

**Figure 4:**
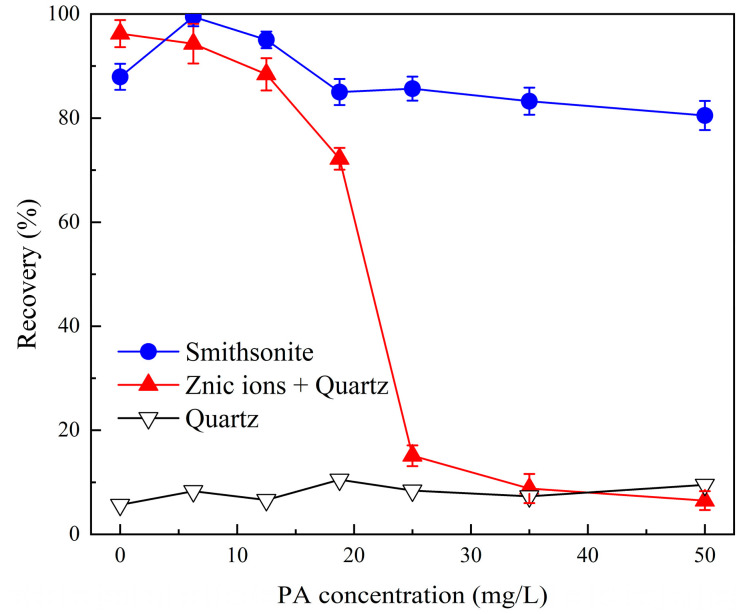
Flotation recovery of smithsonite and quartz at various phytic acid dosages, (C_(NaOL)_ = 120 mg/L, C_(Zn ions)_ = 1 × 10^−4^ mol/L, pH = 10).

Using NaOL as the collector, microflotation recovery of Zn^2+^-activated quartz and smithsonite was tested to evaluate the effect of phytic acid on the flotation recovery of Zn^2+^-activated quartz and smithsonite. The influence of Zn^2+^ dosage on the flotation recovery of smithsonite and quartz is displayed in Figure 2. Based on these findings, the flotation recovery of the minerals with and without the addition of phytic acid at various pH values (Figure 3) and various phytic acid dosages (Figure 4) was studied. After determining the pH and phytic acid dosage, the separation experiment of artificial mixed minerals was performed to determine the influence of phytic acid on flotation separation.

As can be seen in Figure 2, at a Zn^2+^ dosage of 0 mol/L, the flotation recoveries of smithsonite and bare quartz were 90.42% and 5.25%, respectively, indicating that it was reasonably simple to separate smithsonite from pure quartz. With increasing Zn^2+^ dosage, the flotation recovery of smithsonite was higher than 86%, indicating that Zn^2+^ did not affect the flotation behavior of smithsonite. However, Zn^2+^ considerably changed the flotation behavior of quartz. Increasing the Zn^2+^ concentration significantly increased the flotation recovery of quartz. When the Zn^2+^ dosage was 1.5 × 10^−4^ mol/L, the recovery reached 96.24%, indicating that the floatability of quartz was activated by Zn^2+^, thereby posing a challenge in the flotation separation of the minerals. Hence, the use of highly selective depressants in the flotation separation of smithsonite and Zn^2+^-activated quartz should be investigated.

Figure 3 shows the flotation recovery of smithsonite, bare quartz, and Zn^2+^-activated quartz with and without the addition of 35 mg/L phytic acid at various pH values. Within pH 7–12, the floatability of bare quartz without Zn^2+^ was poor, and flotation recovery was below 7%. However, upon the addition of Zn^2+^, the quartz recovery increased with increasing pulp pH. The highest flotation recovery, approximately 96.24%, was achieved at pH 10. This improvement in flotation recovery may be attributed to the influence of pH on the deposition of Zn^2+^. Within the pH range of 9–12, the difference in flotation recovery between smithsonite and Zn^2+^-activated quartz was small. Consequently, separating these minerals through flotation alone without using a depressant would be challenging.

As shown in Figure 3b, the flotation recovery of quartz pretreated with Zn^2+^ was below 12% at pH < 10, indicating that the introduction of phytic acid significantly affected the flotation recovery of Zn^2+^-pretreated quartz. At this pH range, the flotation recovery of quartz pretreated with Zn^2+^ decreased to a level comparable to that of pure quartz. At pulp pH > 10, the flotation recovery of quartz pretreated with Zn^2+^ increased with increasing pH, indicating that the inhibitory effect of phytic acid decreased at high pH. Phytic acid had only a minor effect on smithsonite flotation recovery and thus can be used to inhibit Zn^2+^-pretreated quartz during the flotation of smithsonite.

Figure 4 illustrates the flotation recovery of smithsonite and quartz at various phytic acid dosages. Within the dosage range of 0 to 50 mg/L, the flotation recovery of bare quartz was consistently below 10%. The flotation recovery of quartz pretreated with phytic acid quickly decreased with increasing phytic acid dosage. When the phytic acid dosage was 35 mg/L, the recovery was 9.80%. The flotation recovery of smithsonite did not change when the phytic acid dosage was below 12.5 mg/L but decreased slightly with a further increase in phytic acid concentration. These findings suggest that phytic acid possesses an effective selective inhibitory influence on Zn^2+^-activated quartz. In theory, smithsonite and Zn^2+^-activated quartz could be effectively separated using 35 mg/L phytic acid.

To confirm the potential of phytic acid in the flotation separation of smithsonite from Zn^2+^-activated quartz, artificial mixed mineral flotation tests were carried out. Smithsonite and quartz were combined in a 1:1 mass ratio to create the artificial mixed mineral, with a Zn content of 25.26%. As shown in Table 1, without the addition of phytic acid, the yield of concentrate was as high as 93.74%, and the zinc grade of the concentrate was 25.21%, which is equivalent to that of the feed. This finding indicates that a large amount of quartz entered the concentrate, making the selective separation extremely challenging. The addition of 35 mg/L phytic acid increased the Zn grade in the concentrate from 25.21% to 27.11%, revealing that a good flotation separation was still not achieved. However, the addition of 75 mg/L phytic acid reduced the yield of the concentrate and significantly improved the Zn grade. The Zn grade of the concentrate was 41.98%, and the recovery was 85.41%. Hence, using phytic acid as a depressant could result in excellent flotation separation of smithsonite from Zn^2+^-activated quartz. In addition, the concentration of phytic acid necessary to accomplish the optimal separation of artificial mixed minerals was approximately twice the predicted concentration, which could be attributed to the presence of dissolved Zn ions on the smithsonite surface.

### 2.2. Effect of Phytic Acid on the Zeta Potentials of Minerals

**Figure 5:**
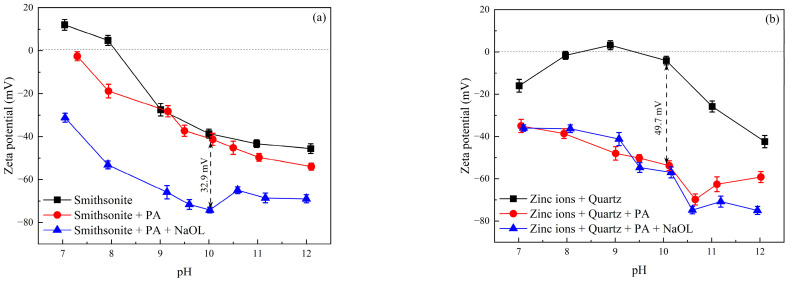
Zeta potential of smithsonite and Zn^2+^-activated quartz at various pH levels with and without the addition of phytic acid or phytic acid + sodium oleate. (**a**) Zeta potential of smithsonite; and (**b**) zeta potential of Zn^2+^-activated quartz, (C_(PA)_ = 35 mg/L), C_(NaOL)_ = 120 mg/L).

The zeta potentials of Zn^2+^-activated quartz and smithsonite were measured by varying the solution pH with and without the introduction of phytic acid or phytic acid + sodium oleate, and the results are presented in Figure 5. Similar to previous findings, the isoelectric point of bare smithsonite was about pH 8.0 [19,20], as depicted in Figure 5a. The zeta potentials of smithsonite and smithsonite pretreated with 35 mg/L phytic acid decreased with increasing pH. The pretreatment with phytic acid resulted in a slightly negative shift in the zeta potential at pH > 9, indicating that phytic acid was rarely adsorbed on the smithsonite surface. When sodium oleate was introduced into the solution, the zeta potential significantly decreased, suggesting that phytic acid pretreatment barely affected the adsorption of sodium oleate onto the smithsonite surface. Therefore, the presence of phytic acid would not hinder the subsequent adsorption of sodium oleate on the smithsonite surface and would have minimal inhibitory effects on the flotation of smithsonite.

The zeta potentials of Zn^2+^-activated quartz initially increased and then decreased as the pH increased, with the highest value at pH 9. The zeta potentials of Zn^2+^-activated quartz pretreated with phytic acid decreased considerably within the entire pH range, indicating that phytic acid may be adsorbed on the Zn^2+^-activated quartz surface, thereby affecting the surface properties. When sodium oleate was introduced into the pulp, the zeta potentials decreased slightly at pH > 9.5. Hence, sodium oleate had minimal adsorption on the surface of Zn^2+^-activated quartz pretreated with phytic acid. Phytic acid depressed the flotation of quartz activated by Zn^2+^ when sodium oleate was used as the collector.

### 2.3. Effect of Phytic Acid on the Migration of Active Zinc Ions

**Figure 6:**
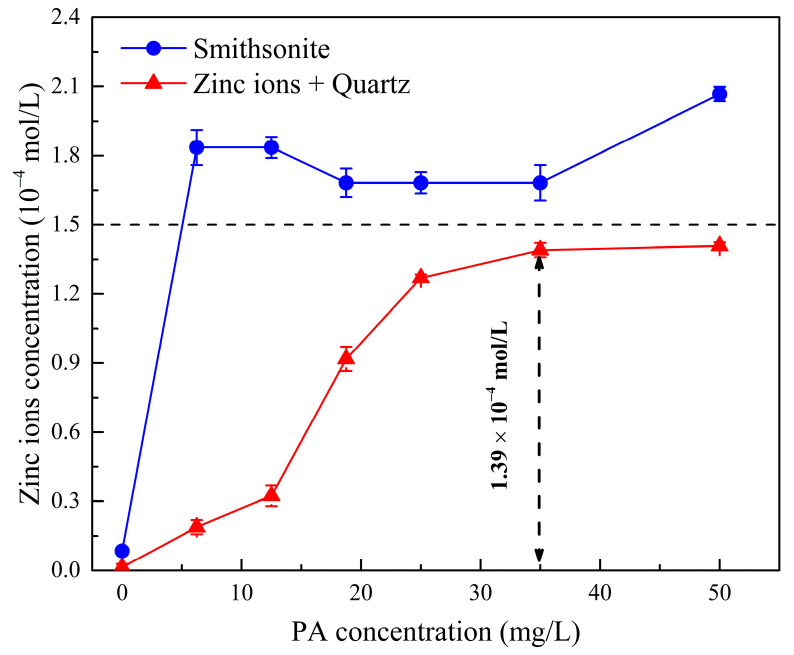
Zn^2+^ concentration dissolved in smithsonite and Zn^2+^-activated quartz solutions at various phytic acid dosages, (pH = 10).

The effect of phytic acid on the migration of active Zn^2+^ was investigated by dissolution tests and scanning electron microscopy combined with energy-dispersive X-ray spectroscopy (SEM–EDS) analysis. The amount of dissolved Zn^2+^ in Zn^2+^-activated quartz and smithsonite solutions was measured at various phytic acid dosages, and the results are shown in Figure 6. The amounts of Zn^2+^ dissolved from the surface of Zn^2+^-activated quartz and smithsonite without phytic acid were 1.3 × 10^−6^ and 8.5 × 10^−6^ mol/L, respectively. The small amount of Zn^2+^ dissolved from the mineral surface had a negligible effect on flotation. By contrast, the amount of Zn^2+^ dissolved from the smithsonite surface increased significantly and then remained constant after the introduction of more phytic acid, indicating that the dissolution of Zn^2+^ from the smithsonite surface reached equilibrium quickly. This finding may explain the need to increase the amount of phytic acid to obtain satisfactory flotation separation in artificial mixed mineral tests.

For Zn^2+^-activated quartz, the Zn^2+^ dissolution increased with increasing phytic acid dosage up to 35 mg/L and did not change when the phytic acid dosage exceeded 35 mg/L. At this phytic acid dosage, the residual Zn^2+^ dosage on the quartz surface was only 1.1 × 10^−5^ mol/L, which was about 7.2% of the added dosage of Zn ions. The Zn^2+^-activated quartz surface lost a significant amount of Zn active sites, which reduced the number of active sites available for sodium oleate adsorption on the activated mineral surface and, consequently, flotation recovery.

**Figure 7:**
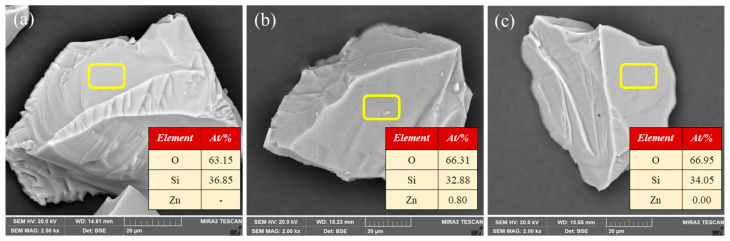
SEM-EDS analysis of quartz samples. (**a**) Bare quartz; (**b**) Zn^2+^-activated quartz; and (**c**) Zn^2+^-activated quartz + phytic acid, (C_(Zn ions)_ = 1 × 10^−4^ mol/L, C_(PA)_ = 35 mg/L, pH = 10).

SEM-EDS analysis was performed to determine the effect of PA on the migration of Zn^2+^ on the quartz surface. Figure 7 shows the micrographs and energy dispersive analyses of the quartz samples with and without pretreatment using Zn^2+^ or Zn^2+^ + phytic acid. The pure quartz had a clean and smooth surface (Figure 7a), and only Si and O were observed, indicating that the quartz sample was uncontaminated.

Upon the addition of Zn^2+^, tiny particles were observed on the surface (Figure 7b), and the presence of 0.80% Zn was detected alongside Si and O. The formation of a Zn-containing mineral surface could be due to the migration of Zn^2+^ from the solution to the quartz surface. Importantly, the introduction of phytic acid restored the apparent morphology of the quartz surface (Figure 7c), and the Zn content was significantly reduced to 0.00%. These results suggest that phytic acid dissolved the Zn species that precipitated on the quartz surface. The depression mechanism of phytic acid on Zn^2+^-activated quartz possibly involves the desorption of active Zn^2+^ from the quartz surface.

### 2.4. XPS Analysis Results

**Figure 8:**
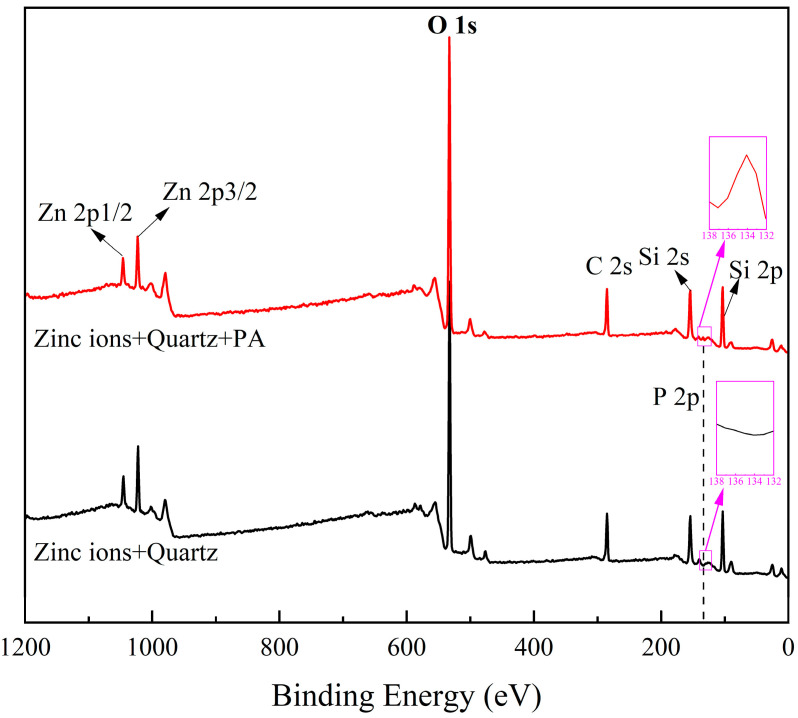
XPS survey spectra of Zn^2+^-activated quartz with and without phytic acid.

**Figure 9:**
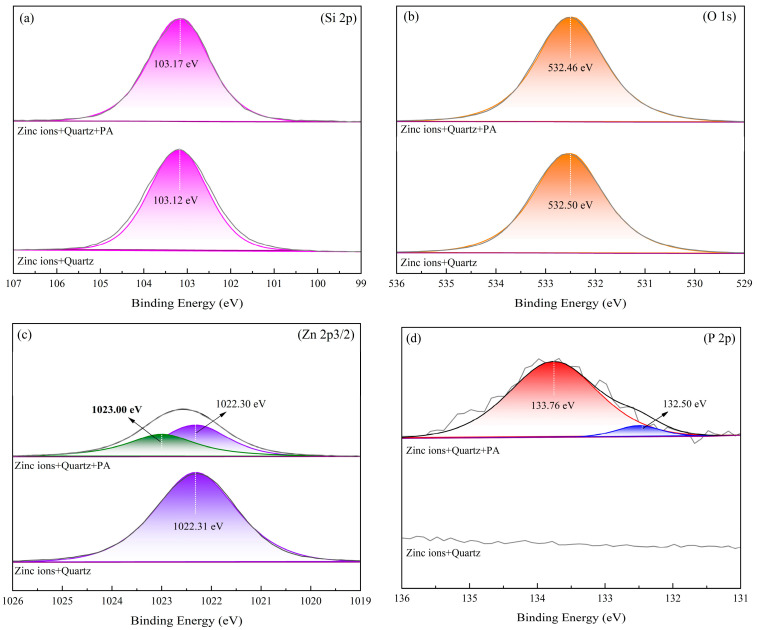
High-resolution XPS spectra of Zn ion-activated quartz and Zn ion-activated quartz + phytic acid. (**a**) Si 2p; (**b**) O 1s; (**c**) Zn 2p3/2; and (**d**) P 2p.

XPS analyses of Zn^2+^-activated quartz with and without phytic acid treatment (35 mg/L) were carried out at pH = 10 to study the adsorption mechanism of phytic acid on the quartz surface treated with Zn ions. The atomic concentrations of the samples with and without phytic acid pretreatment are shown Table 2. The XPS survey spectra and high-resolution spectra of Si 2p, O 1s, Zn 2p^3/2^, and P 2p are depicted in Figure 8 and Figure 9, respectively.

Figure 8 shows the spectral peaks of Zn, Si, and O on the surface of Zn ion-activated quartz, with concentrations of 5.14%, 29.41%, and 65.45%, respectively (Table 2). After the phytic acid treatment, a new spectral peak of P with a concentration of 0.78% appeared, and the Zn atomic concentration decreased to 1.09%. Hence, the introduction of PA changed the properties of Zn^2+^-activated quartz surface.

The high-resolution spectra of Si 2p and O 1s of Zn^2+^-activated quartz in Figure 9 were fitted reasonably with binding energies of 103.12 and 532.50 eV, respectively, which correspond to the presence of quartz. After the pretreatment of phytic acid, the spectra of Si 2p and O 1s were fitted with binding energies of 103.17 and 532.46 eV, respectively. These results suggest that phytic acid had little effect on the Si 2p and O 1s chemical states of the sample. However, the treatment of phytic acid shifted the peaks of the Zn 2p^3/2^ spectra of the Zn^2+^-activated quartz, indicating that the chemical environment of Zn was altered by the treatment. The peak of the Zn 2p^3/2^ spectrum of Zn^2+^-activated quartz without phytic acid was fitted at 1022.31 eV, indicating the presence of Zn-OH [9,21]; hence, the quartz surface was adsorbed by Zn hydroxide species. After treatment with phytic acid, the intensity of the peak decreased, and two spectra with peaks at 1022.30 and 1023.00 eV emerged, which could be attributed to the Zn 2p^3/2^ peak of Zn-PO_4_^3−^ [22]. In addition, the characteristic peak of P 2p appeared on the surface after the addition of phytic acid, indicating the adsorption of the acid on the mineral surface. This P 2p characteristic peak can be separated into two peaks at energies of 132.50 and 133.75 eV, which represent the –PO_3_^2−^ of the phytic acid molecule and Zn-O-P group, respectively [17,22]. Hence, phytic acid could chemisorb with Zn sites on the surface of Zn^2+^-activated quartz.

## 3. Materials and Methods

### 3.1. Single Smithsonite and Quartz Sample and Reagents

Hand-picked and hand-crushed smithsonite and quartz samples were rinsed several times with deionized water. After the samples had been desiccated, they were ground using a planetary ball mill. The ground fine mineral particles were then sieved to produce various size fractions. 38–74 μm smithsonite and quartz samples were used for microflotation. And −2 μm samples were used for XRD analysis, zeta potential test, and XPS analysis. The single mineral samples of quartz and smithsonite contain 99% SiO_2_ and 96% ZnCO_3_, respectively. The XRD patterns of the quartz and smithsonite can be seen in Reference [2], which further indicates that the used single smithsonite and quartz samples are of high purity.

ZnSO_4_ used in this study was analytically pure and was used to pretreat quartz. Phytic acid (C_6_H_18_O_24_P_6_) was also analytically pure and used to inhibit the flotation of quartz. Sodium oleate (NaOL, chemically pure) was employed to float the minerals. To modify and maintain the pulp pH levels, analytically pure HCl and NaOH were used. All solutions and pulp used in this study were prepared with deionized water. 

### 3.2. Microflotation Tests

Microflotation investigations were conducted using an inflatable hanging slot flotation device (XFG). For a test, 2.0 g of single mineral (or artificial mixed minerals) was put into a 40 mL cell containing a certain amount of distilled water [22]. First, the pulp pH was modified by hydrochloric acid or sodium hydroxide and stirred for 3 min. Second, if necessary, ZnSO_4_ and phytic acid were added in succession to the pulp while stirring for 3 and 5 min, respectively. Then, the mineral suspension was conditioned for 3 min after sodium oleate was added. Diluted sodium hydroxide or hydrochloric acid was used to maintain the suspension pH at an acquired value throughout the entire conditioning process. Finally, 3 mins of flotation was performed. The flotation recovery of single mineral test was obtained by filtering, drying, and weighing both the concentrate and tailing samples. The Zn recovery of artificial mixed minerals was calculated based on the weight and the grade of zinc of the concentrate and tailing. The Zn grades of the products were analyzed by XRF.

### 3.3. Zeta Potential Measurements

A Coulter Delsa 440sx zeta analyzer (Beckman Coulter, Brea, CA, USA) was used to perform zeta potential measurement. A KCl solution (1 × 10^−3^ mol/L) was used as a supporting electrolyte. A total of 30 mg of the −2 μm sample was used for each test. The pulp preparation process for the test was identical to that of the microflotation investigation. Zeta potential was measured using the supernatant liquor after the pulp settled for 10 min. At each reagent condition, at least three independent tests were performed.

### 3.4. Zinc Ions Dissolution Tests and SEM-EDS Analysis

The amount of Zn^2+^ dissolved in the smithsonite and Zn^2+^-activated quartz solutions was determined using an inductively coupled plasma spectrometer optical emission spectrometer (ICP-AES, Spectro Blue II-SOP, Kleve, Germany). For smithsonite, 2.0 g of sample was added into a 40 mL solution and stirred with a magnetic mixer for 5 min before being treated with various dosages of PA and stirred for another 15 min at pH 10. For quartz, 1.5 × 10^−4^ mol/L Zn^2+^ was put into the solution and mixed for 5 min, and then desired dosage of phytic acid was added and stirred for another 15 min at pH 10. For the test, the supernatant was collected after centrifugation.

Scanning electron microscopy combined with energy dispersive X-ray spectroscopy (SEM–EDS) (AMETEK, Inc., Berwyn, PA, USA) was employed to analyze the surface property of quartz samples. First, a desired quartz sample was stirred for 15 min in a solution containing zinc ions or zinc ions + phytic acid at pH 10. After solid–liquid separation, the solid powder was placed in a vacuum drying oven (40 °C, 2 h). Before conducting the SEM-EDS analysis, the samples were sprayed with gold to enhance their conductivity.

### 3.5. XPS Analysis

An ESCALAB 250Xi XPS (Thermo Fisher, Waltham, MA, USA) spectrometer with a monochromatic Al K-ray source at 150 W was used to determine the atomic concentrations of Si, O, Zn, and P and their chemical states of quartz. For the XPS analyses, suspensions of Zn^2+^-activated quartz (0.5 g quartz, 1.5 × 10^−4^ mol/L Zn^2+^) with and without the pretreatment of phytic acid (35 mg/L, pH = 10) were stirred for 30 min. The solid powder was obtained by centrifugation. And a vacuum drying oven was used to dry the powder for the test.

## 4. Conclusions

The following conclusions are drawn based on the experimental results of the microflotation test; zeta potential measurement; and ICP-AES, SEM-EDS, and XPS analyses:

1. The addition of 35 mg/L phytic acid selectively inhibited the flotation of Zn^2+^-activated quartz without affecting the flotation of smithsonite, enabling the separation of these minerals.
2. A high dosage of phytic acid (75 mg/L) was required to achieve satisfactory separation of artificially mixed minerals.
3. Phytic acid exhibited a chemisorption capability with the Zn sites on the surface of Zn^2+^-activated quartz, resulting in the desorption of Zn ions from the quartz surface into the solution.
4. The significant desorption of Zn ions from the mineral surface reduced the adsorption sites available for sodium oleate, leading to a significant decrease in the flotation recovery of Zn^2+^-activated quartz.
5. Phytic acid had minimal influence on the adsorption of sodium oleate and the flotation recovery of smithsonite. Therefore, a satisfactory flotation separation of smithsonite from Zn^2+^-activated quartz was achieved.

These findings demonstrated the effective role of phytic acid as a selective depressant for Zn^2+^-activated quartz in the flotation of smithsonite when sodium oleate was used as the collector.

## Figures and Tables

**Table 1:** Flotation results of artificial mixed mineral flotation, (pH = 10, C_(NaOL)_ = 120 mg/L).

Phytic Acid Concentration (mg/L)	Product	Yield (%)	Zn Grade (%)	Zn Recovery (%)
0	Concentrate	93.74	25.21	93.55
	Tailing	6.26	26.03	6.45
	Feed	100.00	25.26	100.00
35	Concentrate	85.12	27.11	91.18
	Tailing	14.88	15.00	8.82
	Feed	100.00	25.31	100.00
75	Concentrate	51.37	41.98	85.41
	Tailing	48.63	7.58	14.59
	Feed	100.00	25.25	100.00

**Table 2:** Atomic concentrations of Zn^2+^-activated quartz with and without phytic acid.

Sample	Atomic Concentrations (%)			
	Si 2p	O 1s	Zn 2p	P 2p
Zn^2+^-activated quartz	29.41	65.45	5.14	-
Zn^2+^-activated quartz + phytic acid	31.85	66.28	1.09	0.78

## Data Availability

Not applicable.
